# Soil bacterial community profiling of seed potato plantation sites in Taiwan

**DOI:** 10.1128/mra.01363-25

**Published:** 2026-02-13

**Authors:** Yung-Hao Tung, Dao-Yuan Xue, Yu-Shen Chen, Shih-Min Su, Yen-Hsin Chiu, Yuan-Min Shen

**Affiliations:** 1 Department of Plant Pathology and Microbiology, National Taiwan University 33561, Taipei, Taiwan; 2 Taiwan Seed Improvement and Propagation Station, Ministry of Agriculture 56091 , Taichung, Taiwan; 3 Master Program for Plant Medicine, National Taiwan University 33561, Taipei, Taiwan; 4 Plant Teaching Hospital, National Taiwan University 33561, Taipei, Taiwan; DOE Joint Genome Institute, Berkeley, California, USA

**Keywords:** bacterial community, microbiota, soil microbiology, potato, vegetable

## Abstract

Potato (*Solanum tuberosum* L.) is one of the major food sources globally. This study collected soil samples from multiple seed potato cultivation sites in Taiwan and employed amplicon sequencing to analyze microbial community composition. A total of 6,597 amplicon sequence variants of bacteria were identified and belonged to 75 classes.

## ANNOUNCEMENT

**Fig 1:**
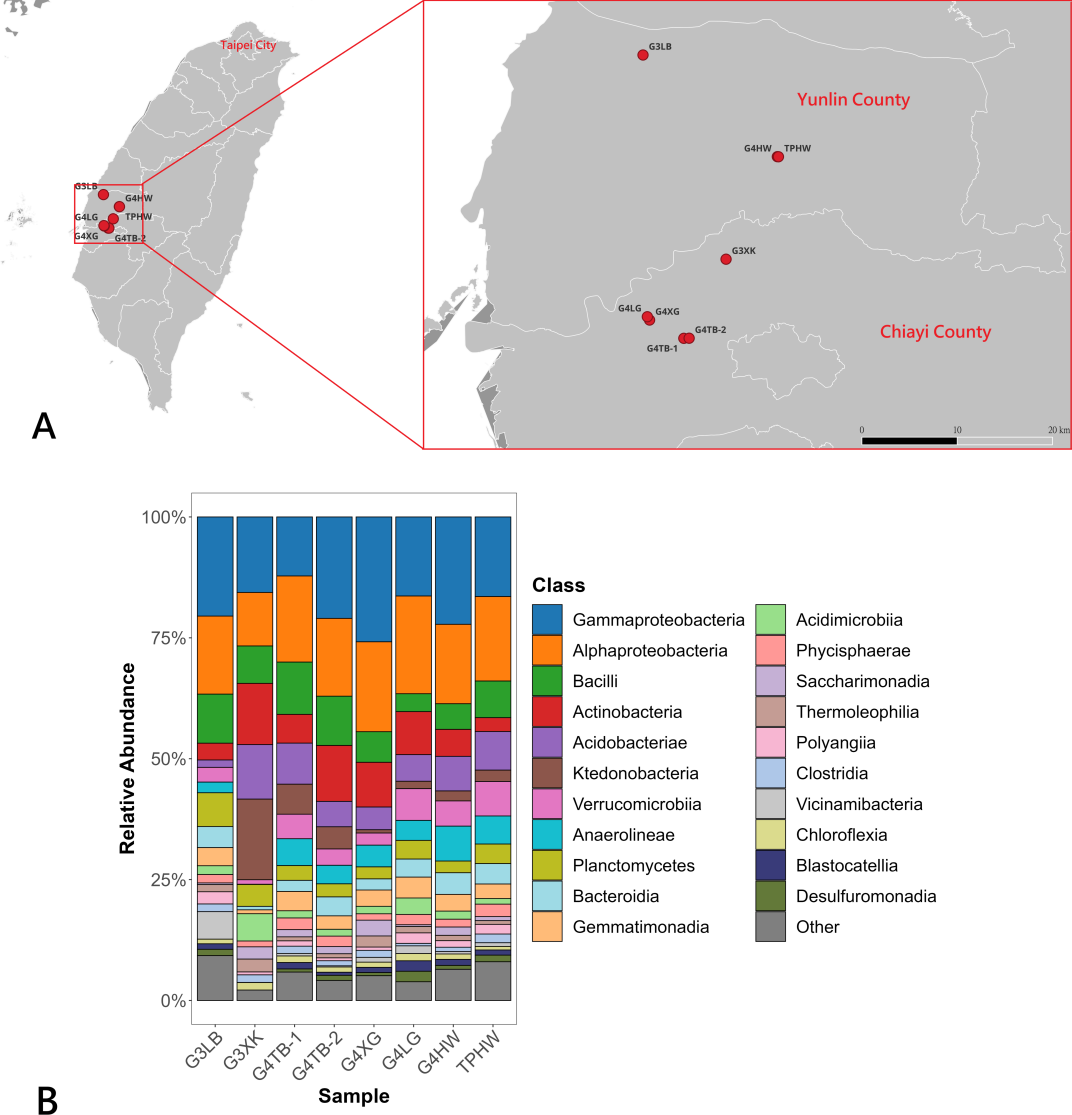
(**A**) Location of sampling sites in Taiwan, including potato planting areas in Yunlin County and Chiayi County. The map was constructed using free licensed software (QGIS, Open Source Geospatial Foundation, version 3.40.6), with map layouts provided under the Open Government Data License by the National Land Surveying and Mapping Center, Ministry of the Interior, Taiwan (version 1140318). (**B**) Relative abundance of soil bacterial communities at the class level, displaying classes with an average abundance >1%.

Potato (*Solanum tuberosum* L.) is one of the world’s major food crops. It is typically propagated vegetatively by replanting seed tubers from previous harvests in the subsequent growing season (1). Previous studies have shown that the potato-associated microbiome can significantly influence plant health (2). In this study, we collected soil samples from several seed potato cultivation sites in Taiwan, including fields participating in the healthy seed potato certification program (3). Using amplicon sequencing, we characterized the microbial community composition to enhance our understanding of the microorganisms inhabiting seed potato cultivation environments. Eight soil samples were collected from potato cultivation sites in Yunlin and Chiayi Counties, Taiwan, during January and February 2024 (Table 1; Fig. 1). The sampling sites included two fields growing certified generation 3 seed potatoes (G3LB and G3XK) in greenhouses, five fields growing generation 4 seed potatoes (G4TB-1, G4TB-2, G4XG, G4LG, and G4HW), and one field producing table potatoes (TPHW). At the time of sampling, all potato plants had been cultivated for some time and were approximately 1–2 weeks before harvest. The top 5 cm of surface soil was removed, and approximately 500 g of soil adjacent to potato plants was collected using a clean shovel and placed into sterile ziplock bags. The shovel was disinfected with 75% ethanol between collections. All samples were transported to the laboratory on the day of collection at room temperature. Approximately 50 mL of each soil sample was transferred into centrifuge tubes and stored at −80°C until DNA extraction.

**TABLE 1:** Soil sample collection and amplicon sequencing summary

Sample ID	Sampling date	Sampling location	Sample coordinates	No. of raw reads	Filtered forward read	SRA accession no.
G3LB	30 Jan. 2024	Lunbei Township, Yunlin County	23°47′37.9″N 120°18′07.6″E	64,555	50,659	SRR35927175
G3XK	15 Feb. 2024	Sikou Township, Chiayi County	23°35′02.3″N 120°23′16.9″E	62,147	42,255	SRR35927172
G4TB-1	30 Jan. 2024	Taibao City, Chiayi County	23°30′09.5″N 120°20′38.6″E	67,761	53,486	SRR35908604
G4TB-2	30 Jan. 2024	Taibao City, Chiayi County	23°30′09.9″N 120°20′58.3″E	69,696	54,641	SRR35908603
G4XG	30 Jan. 2024	Singang Township, Chiayi County	23°31′17.2″N 120°18′31.9″E	66,532	53,231	SRR35927177
G4LG	30 Jan. 2024	Lioujiao Township, Chiayi County	23°31′29.4″N 120°18′23.6″E	62,244	50,008	SRR35927176
G4HW	30 Jan. 2024	Huwei Township, Yunlin County	23°41′21.7″N 120°26′24.7″E	78,533	62,830	SRR35927174
TPHW	30 Jan. 2024	Huwei Township, Yunlin County	23°41′21.4″N 120°26′28.7″E	64,705	50,871	SRR35927173

Soil DNA was extracted using the Geneaid Presto Soil DNA Extraction Kit (Cat. No. SLD050; Geneaid, Taiwan) following the manufacturer’s instructions. The V3–V4 region of the bacterial 16S rRNA gene was amplified using primers 341F (5′-CCTACGGGNGGCWGCAG-3′) and 785R (5′-GACTACHVGGGTATCTAATCC-3′) (4). Sequencing libraries were prepared using the TruSeq DNA PCR-Free Sample Preparation Kit (Illumina, USA) according to the manufacturer’s protocol, with index codes added for sample identification. Amplicon sequencing was performed on the Illumina MiSeq platform (Biotools, Taiwan) to generate sequence reads. A total of 536,173 forward reads were processed using the DADA2 pipeline (version 1.30.0) (5). Primer and low-quality reads were removed using the parameters maxN = 0, maxEE = 2, and truncQ = 2. After denoising, amplicon sequence variants (ASVs) predominantly with a length of 283 bp were obtained. Taxonomy was assigned using the RDP naive Bayesian classifier in DADA2, with the SILVA database as the reference (version 138.2) (6).

A total of 6,597 bacterial ASVs were identified, representing 75 classes. Among these, 21 classes had an average relative abundance greater than 1%, including Gammaproteobacteria (18.8%), Alphaproteobacteria (16.7%), Bacilli (7.7%), Actinobacteria (7.5%), Acidobacteriae (6.5%), Ktedonobacteria (4.3%), Verrucomicrobiia (4.2%), Anaerolineae (4.2%), Planctomycetes (3.8%), Bacteroidia (3.3%), Gemmatimonadia (3.2%), Acidimicrobiia (2.3%), Phycisphaerae (1.9%), Saccharimonadia (1.5%), Thermoleophilia (1.4%), Polyangiia (1.4%), Clostridia (1.3%), Vicinamibacteria (1.3%), Chloroflexia (1.2%), Blastocatellia (1.1%), and Desulfuromonadia (1.0%). The remaining 5.6% of sequences were assigned to classes with <1% relative abundance. This characterization serves as a reference for comparing soil bacterial community structure among different potato production programs, including certified seed potato cultivation.

## Data Availability

The 16S rRNA gene raw amplicon sequence data from this study have been deposited in GenBank SRA under accession numbers SRR35908603–SRR35908604/SRR35927172–SRR35927177 and BioProject accession number PRJNA1354991.
